# Bt Crop Effects on Functional Guilds of Non-Target Arthropods: A Meta-Analysis

**DOI:** 10.1371/journal.pone.0002118

**Published:** 2008-05-07

**Authors:** L. LaReesa Wolfenbarger, Steven E. Naranjo, Jonathan G. Lundgren, Royce J. Bitzer, Lidia S. Watrud

**Affiliations:** 1 Department of Biology, University of Nebraska at Omaha, Omaha, Nebraska, United States of America; 2 USDA-ARS Arid Land Agricultural Research Center, Maricopa, Arizona, United States of America; 3 USDA-ARS North Central Agricultural Research Laboratory, Brookings, South Dakota, United States of America; 4 Department of Entomology, Iowa State University, Iowa, United States of America; 5 U.S. Environmental Protection Agency, National Health and Environmental Effects Research Laboratory, Western Ecology Division, Corvallis, Oregon, United States of America; University of Pretoria, South Africa

## Abstract

**Background:**

Uncertainty persists over the environmental effects of genetically-engineered crops that produce the insecticidal Cry proteins of *Bacillus thuringiensis* (Bt). We performed meta-analyses on a modified public database to synthesize current knowledge about the effects of Bt cotton, maize and potato on the abundance and interactions of arthropod non-target functional guilds.

**Methodology/Principal Findings:**

We compared the abundance of predators, parasitoids, omnivores, detritivores and herbivores under scenarios in which neither, only the non-Bt crops, or both Bt and non-Bt crops received insecticide treatments. Predators were less abundant in Bt cotton compared to unsprayed non-Bt controls. As expected, fewer specialist parasitoids of the target pest occurred in Bt maize fields compared to unsprayed non-Bt controls, but no significant reduction was detected for other parasitoids. Numbers of predators and herbivores were higher in Bt crops compared to sprayed non-Bt controls, and type of insecticide influenced the magnitude of the difference. Omnivores and detritivores were more abundant in insecticide-treated controls and for the latter guild this was associated with reductions of their predators in sprayed non-Bt maize. No differences in abundance were found when both Bt and non-Bt crops were sprayed. Predator-to-prey ratios were unchanged by either Bt crops or the use of insecticides; ratios were higher in Bt maize relative to the sprayed non-Bt control.

**Conclusions/Significance:**

Overall, we find no uniform effects of Bt cotton, maize and potato on the functional guilds of non-target arthropods. Use of and type of insecticides influenced the magnitude and direction of effects; insecticde effects were much larger than those of Bt crops. These meta-analyses underscore the importance of using controls not only to isolate the effects of a Bt crop *per se* but also to reflect the replacement of existing agricultural practices. Results will provide researchers with information to design more robust experiments and will inform the decisions of diverse stakeholders regarding the safety of transgenic insecticidal crops.

## Introduction

Meeting future food, feed and fiber needs without compromising environmental integrity is a central challenge for agriculture globally [1]. Growers throughout the world are rapidly adopting genetically-engineered (GE) crops with 102 million hectares produced globally in 2006 [2]. About a third of this production involves cotton and maize plants that have been engineered to produce one or more insecticidal proteins (Cry toxins) from the common soil microbe *Bacillus thuringiensis* Berliner (Bt) for control of lepidopteran and coleopteran pests. The potential environmental impact of these insect-resistant GE crops has been debated vigorously with most of the focus on non-target organisms, and to a lesser extent, their associated ecosystem function [3]–[8]. Currently, our understanding of the impacts of Bt crops on ecological function is limited because with few exceptions [9]–[11], individual and review studies have focused almost exclusively on the taxonomic level (e.g. species, families, order). While researchers, regulators and policy-makers recognize the need to understand impacts of Bt crops on ecological function and associated ecosystem services such as biological pest control, these issues require the synthesis and interpretation of many studies on a diverse group of species. Such a synthesis is precluded in individual studies because the number of taxonomic groups examined is limited, thus confounding ecological function and taxonomy. Here, we report the first synthesis of Bt crop effects on ecological guilds and their interactions.

Declines in insecticide use are associated with the increasing adoption of Bt maize and cotton [12], and GE crops may have a reduced impact on non-target organisms relative to current pest management practices [11], [13]. Some studies have shown negative impacts on the abundance and life history of charismatic and beneficial species (e.g., [14]–[16]), leaving questions about whether GE crops have minimal ecological effects. A recent meta-analysis [13] provided a broad synthesis on how Bt cotton and maize alter the abundance of non-target arthropods as a combined group. In particular, the effect of Bt crops varied from negative to positive depending on what pest management practices were compared. As a single pest management strategy (i.e., no insecticide use), Bt crops reduced the abundance of non-target organisms as a group compared to using no pest management interventions, but increased abundance when Bt crops substituted for insecticides. When Bt and insecticides are used together as a strategy compared to only insecticides, there was no consistent change in the abundance of non-target arthropods. Taxonomic-specific effects on non-target orders were detected when comparing the use of Bt crops to using no pest management tactics.

While previous analyses contribute substantially to understanding the impacts on species and taxa, we have lacked a broader perspective of how Bt crops may affect ecological functions of the complex insect communities associated with agroecosystems. Arthropods within agroecosystems provide numerous ecological services and economic benefits to land managers. Predators, omnivores, and parasitoids consume insect pests and weed seeds [17]–[21]; detritivores aid in degrading crop residue and improve soil health [22], [23]; and herbivores can reduce competition by non-crop plants and serve important roles as prey and hosts for natural enemies [24]. These services and others in natural and managed habitats amount to an estimated $57 billion annually [25]. Because these functional guilds interact differently with crop plants and environments, they are likely to be affected by pest management practices to varying degrees. Thus, a comprehensive examination that simultaneously accounts for different crop production systems and pest management practices is required to draw meaningful conclusions about the environmental impact of Bt crops.

Here, we utilize a modified subset of the 171-study non-target database compiled by Marvier et al. [13] to analyze the effects of Bt crops on the abundance of non-target functional guilds of arthropods within agroecosystems. Using meta-analysis, we examine how Bt cotton, Bt maize and Bt potato affect the abundance of predators, parasitoids, omnivores, detritivores and herbivores, and the relationships between predators and herbivores and between predators and detritivores in field studies. We extensively evaluate the robustness of the metadata set relative to experimental design factors (e.g., plot size, numbers and times of sampling), publication bias, and type of Bt toxins examined. When sample sizes allow, we also provide information about several common species within these functional guilds. The magnitude and direction of ecological effects of Bt crops on non-target arthropods were all examined and interpreted as a sole pest management strategy and within the context of current pest management practices.

## Methods

### Searching

We used a modified subset of the full meta-dataset discussed by Marvier et al. [13] that is available at http://delphi.nceas.ucsb.edu/btcrops. Briefly, the studies in the full dataset include 1) field crops that were genetically modified to express one or more Cry proteins from *B. thuringiensis*, 2) studies that measured the effect of the GE crop on abundance or other attributes of non-target arthropod taxa relative to a non-transgenic control, 3) studies that reported means accompanied by standard deviations (or standard error) and sample size and 4) were published in English. Our analyses were restricted to field studies measuring arthropod abundance in cotton, maize and potato. There were not sufficient studies to directly examine biodiversity or natural enemy function. Our database included studies conducted between 1992 and early 2006.

### Selection

To avoid non-independence issues in our meta-analyses, this field/abundance dataset was further filtered and partitioned. First, three distinct types of comparisons were recognized and analyzed separately. The first set of studies contrasted Bt with non-Bt plots, neither of which received any additional insecticide treatments. This comparison addresses the hypothesis that the toxins in the Bt plant directly or indirectly affect arthropod abundance. It also can be viewed as a comparison between the Bt crop and its associated unsprayed refuge [26]. The second set of studies contrasted unsprayed Bt fields with non-Bt plots that received insecticides. This comparison tests the hypothesis that arthropod abundance is influenced by the method used to control the pest(s) targeted by the Bt crop. The final study type contrasts Bt to non-Bt fields when both are subject to insecticide treatments. The hypothesis tested here is whether arthropod abundance is altered when the Bt crop is not completely effective against the target pest(s) and/or other pests not susceptible to the Bt toxins are problematic [21]. For cotton and potato this represents a more typical commercial practice for both Bt and non-Bt-crops. There were no studies that fell into this final category for Bt maize.

We further eliminated redundant taxonomic categories presented within the same study. For example, a study might include data on individual species and also on pooled taxonomic groups containing these same species. In filtering non-independent data, we always retained the finest taxonomic level possible (e.g., species, genus). Some studies also reported multiple stages of the same species. In these cases we retained the least mobile, but feeding, stage when possible. Thus for example, we retained larvae or nymphs in preference to eggs or adults and retained adults or larvae/nymphs in preference to eggs. Our reasoning was that less mobile stages would experience higher and longer exposure to potential toxins than adults that might be transient residents. Eggs would be the least likely to be exposed. Finally, when studies included measures of both seasonal abundance (averaged over multiple sample dates) and peak abundance (highest density on any given sample date), we retained the seasonal mean. Peak abundances were used only if seasonal mean data were absent. All observations in the database are based on a single season; thus, reported differences in density reflect within-season differences and not cumulative changes over years. In total, the database we used in our analyses contained 2981 observations from 131 experiments reported in 47 published field studies. The database is summarized in Table 1 and is provided as a supplement to this paper (Appendix S1).

**Table 1:** Summary of meta database used in analyses.

Crop Contrast	# of studies	Comparisons per study (range)	Type of Bt toxins	Plot size range (ha)	Study duration range^a^ (days)	Sample dates (range)	Sampling methods^b^	# Taxa^c^	Number of true replicates (range)
**Cotton**									
Non-Bt control	5	1–6	Cry1A, Cry1Ac, Cry1Ab, Cry1Ac+Cry2Aa	0.004–3.7	74–170	1–24	1–3	133	2–12
Non-Bt sprayed	3	3–5	Cry1Ac, Cry1Ac+Cry2Ab	0.06–0.4	41–129	6–24	2–4	29	3–4
Both sprayed	6	1–12	Cry1 Ac, Cry1Ac+Cry2Ab	0.02–17.5	13–123	3–18	2–5	67	3–7
**Maize**									
Non-Bt control	16	1–10	Cry1Ab, Cry3Bb	0.002–13.4	3–732	1–34	1, 2, 5–9	137	2–36
NonBt sprayed	34	1–7	Cry1Ab, Cry3Bb	0.03–2.8	3–732	1–34	2, 4, 5, 6, 8, 9	99	2–16
**Potato**									
Non-Bt control	2	2	Cry3A	0.003–0.03	49–70	15–21	4, 5	21	6
Non-Bt sprayed	2	2	Cry3A	0.003–2.8	49–273	4–21	4, 5	25	6
Both sprayed	2	1	Cry3A	0.05	456–472	5–17	2, 3, 5	18	3

Database includes studies conducted between 1992 and early 2006.

a For the 8 studies conducted multiple years, comparisons were made for each year.

b 1 = vacuum, 2 = plant count, 3 = sweep net, 4 = beat cloth, 5 = pitfall trap, 6 = litter, 7 = pan trap, 8 = soil, 9 = sticky trap

c Based on the finest level of taxonomic resolution provided by study authors; some duplication of taxa may occur if only Family or Order level resolution was provided.

### Data Abstraction

Two additional descriptor columns were added to or modified from the full database of Marvier et al. [13]. The first categorized the non-target organisms into one of six functional guilds (herbivore, omnivore, predator, parasitoid, detritivores, or mixed). These same categories were provided in the original full database, but some of the categorizations were inaccurate (due to subjective factors) or incomplete (we classified all those originally marked as unknown).

We assigned functional categories based on a crop production perspective when ecological function of an organism varied with life stage; e.g., syrphid larvae are predators but adults may be classified as pollinators or herbivores. Although many predators have omnivorous habits, relying on nectar, pollen, and fungal spores or seeds in addition to prey, the ‘omnivore’ category was reserved for those whose diet is generally regarded as equally comprised of prey and non-prey foods (e.g., Formicidae, Elateridae, Gryllidae, some Dermaptera, some Diptera, and some Carabidae). The ‘mixed’ category refers to higher taxonomic groupings (e.g., family, order) where members fell into more than one functional group. A summary of the taxa associated with each functional group is presented in Appendix S2.

Each taxon's feeding style was added to the database to describe the way in which it obtains its food and is potentially exposed to Bt toxins. The categories included piercing, chewing, mandibulate-sucking, rasping, lapping, chewing-lapping and unknown. Most of these categories were derived from the literature [27], [28], numerous other sources and personal experiences of the authors. The ‘unknown’ category applied to both mixed higher taxonomic levels and to life stages known not to feed (e.g., egg and pupal stages).

While both functional guild and feeding style classifications are unavoidably subjective to some degree, every effort was made to designate these species according to the prevailing understanding and knowledge in the entomological community.

### Quantitative data synthesis

All the meta-analyses presented herein use Hedges' *d*, a weighted mean effect size estimator that is calculated as the difference between an experimental (*Bt*) and control (non-*Bt*) mean response divided by a pooled standard deviation and corrected for small sample size bias [29], [30]. In analyses the effect size is weighted by the reciprocal of the sampling variance [29]. The effect size was estimated such that a negative effect size would indicate lower abundance in the *Bt* crop compared with the non-*Bt* control while a positive effect size would indicate higher abundance in the *Bt* crop. All analyses were conducted using MetaWin [30]. For hypothesis testing we primarily used the parametric 95% confidence interval (CI) given the results of normality testing (see below). If the interval enclosed zero, then the effect size was deemed not significantly different from zero. MetaWin also provides bias-corrected, resampling-based estimates of the 95% CI. If parametric and resampled CIs indicated different interpretations, then normality of the comparison was assessed and the appropriate CI was employed.

#### Database robustness and sensitivity analyses

Two general sets of analyses were conducted on our final database. The first evaluated the robustness of the data set and its sensitivity to various factors that could influence the interpretation of results. We tested for publication bias using a weighted histogram of effect sizes [31] and by a funnel plot that diagrams effect size as a function of sample size [32]. To test for data normality, we generated normal quantile plots [33], which present the distribution of effect sizes in the dataset against a normal distribution.

A series of sensitivity analyses were conducted to gauge the effect of 1) various experimental design factors (size of experimental plot, duration of the study, and the total number of sampling dates), 2) individual studies included in the meta-analysis that vary in size, and 3) different Bt toxins produced in the crops. In order to examine any systematic effects of design factors on effect size we conducted weighted (effect size variance) regressions of the absolute values of the effect sizes based on abundances for each design factor. Analyses were conducted on all crops pooled and separately for cotton, maize and potato for each of the control contrasts noted above. To examine sensitivity to individual studies, we conducted a set of functional guild analyses (see below) where each relevant study (depending on crop and control contrast) was eliminated one at a time. Finally, we contrasted lepidopteran-specific versus coleopteran-specific toxins for maize, and the effect of single versus dual gene Bt cottons.

#### Ecological Factors

We used a one-way, fixed effects model to test effect sizes relative to functional guilds. Because functional guild and feeding style were not independent classifications of the taxa examined (Likelihood ratio χ^2^ = 580, *P*<0.01 for cotton; χ^2^ = 1632, P<0.01 for maize; χ^2^ = 72, P<0.01 for potato), we used feeding style to examine heterogeneity within a functional guild or further clarify factors influencing the direction and magnitude of effect sizes. We analyzed functional guilds separately for cotton, maize and potato within each of the three control contrasts. In addition to estimating confidence intervals, MetaWin also estimates total heterogeneity (between-sample variance) and tests its significance with the χ^2^ distribution using *n*-1 degrees of freedom. Significant heterogeneity suggests a non-normal distribution of effect size due to the presence of different subcategories within the functional guild. When significant heterogeneity was indicated, we attempted to partition sources of variation by examining taxonomic groups, and feeding style within functional guilds. We also examined the influence of insecticide type when they were used in control plots. Insecticide data were not available in the database for studies in which both experimental and control plots were sprayed.

#### Predator-nontarget herbivore relationships

We examined community level responses using predator/prey ratios to provide an alternate measure of impact on pest management services. To estimate predator to prey ratios, we identified studies in our database in which both predator and herbivore functional groups were measured. We then summed the mean abundance of predators (*Mean_Predator_*) and herbivores (*Mean_Herbivore_*) for each relevant study and used these measures to estimate the quotient of predators over herbivores (prey). The variance of this quotient is given as
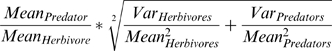
where *Var_Herbivore_* and *Var_Predator_* are the sum of variances of individual herbivores or predators in a given study. We assumed that the covariance between predators and herbivores was zero, and thus this variance estimate is conservative. Species of predators from the studies included were largely generalists so that these ratios reflect general pest management services provided by non-target arthropods. We restricted the designation of prey solely to the herbivore functional group to achieve consistency across studies. Omnivores could function as both predator and herbivores and detritivores are examined in another analysis (see below). We also did not include parasitoids in the ratio. Hedges' *d* effect sizes were calculated from these predator-to-prey quotients and their variance terms. As before, a negative effect size would indicate a lower ratio in the *Bt* crop. The sample size was assumed to be the number of true replicate plots in the experimental design of each study.

#### Predator-detritivore Relationships

We performed a final set of community-level analyses to compare the effects of Bt crops vs. the effects of soil and seed insecticide treatments on detritivores and their predators. We asked whether our meta-analysis would support the results of several single studies (e.g., [34]), in which insecticide applications increased detritivore (particularly collembolan) abundance by reducing their predators. We also asked whether the corresponding effects of Bt crops would be less disruptive. Our analysis included studies in which either or both predators and detritivores were measured.

## Results

### Database Robustness and Sensitivity

There was no indication of publication bias in our dataset. A weighted histogram of effect sizes based on non-target organism abundance in all crops was unimodal and centered on zero. Effect size as a function of sample size was funnel shaped; there were no obvious gaps in distributions at any one sample size, and variation in effect size was characteristically greater at low sample size. Finally, normal quantile plots demonstrated that effect sizes were normally distributed for each of the three crops. We found few significant relationships between absolute effect sizes and experimental design elements, and when differences were noted, slope values were very small. There were no significant relationships between effect size and study duration for all crops pooled or for cotton, maize or potato separately. Effect sizes increased slightly with increasing plot size for maize and all crops pooled but declined slightly in potato when both these Bt crops were compared against sprayed non-Bt controls. Effect sizes declined slightly with increasing numbers of sample dates for maize and all crops combined when the Bt crop was compared to an unsprayed, non-Bt control. Overall, it appears that these experimental design factors had little impact on resulting effect sizes and their interpretation.

Another potential issue with the database is that particular studies may have undue influence on the results, especially those contributing a large number of observations. We assessed this issue by re-running our functional guild analyses following the removal of individual studies. Resulting effect sizes and their interpretations were extremely robust for cotton and maize (72 total study-x-functional-guild analyses in cotton and 204 in corn). For cotton, only two out of 72 analyses (2.8%) resulted in different interpretations. In one case a significant positive effect size, indicating a higher abundance in Bt treatments, became nonsignificant with the elimination of a relatively large study while another changed from nonsignificant to positive with the elimination of a small study. Likewise, in maize only five of 204 (2.4%) analyses differed. Three negative effects, or reduced abundance in Bt treatments, became nonsignificant with the elimination of two large and one medium-sized study, and two positive effects (higher abundance in Bt treatments) became nonsignificant after the elimination of a large and medium study. In contrast, there were only two studies for each of the three control comparisons in potatoes and the elimination of each study in turn resulted in an 11.1% (two of 18 study-x-functional-guild analyses) change in results. Elimination of the largest study resulted in positive effect sizes becoming nonsignificant. Given that Bt potatoes are no longer commercially planted and that we expect no forthcoming studies unless commercial viability changes, we present our results but note their limitations. A synthesis here will provide a starting point for future studies if commercial plantings resume.

A final sensitivity analysis examined responses to different Cry toxins or combination in cotton and maize. Qualitatively, we found no differences in responses by functional guild or all taxa pooled between single (Cry1Ac) and dual (Cry1Ac+Cry2Ab2 or Cry1Ac+Cry2Aa) toxin cotton events. Likewise we found only one qualitative difference in non-target response to lepidopteran-specific (Cry 1Ab, Cry1Ab+Cry1Ac) and coleopteran-specific (Cry3Bb) maize events. The abundance of the parasitoid functional guild (consisting largely of *Macrocentrus* spp.) decreased with the lepidopteran-specific cry protein but not with the coleopteran-specific cry protein (see below for more detail). Due to the strong decrease in abundance in this functional guild in lepidopteran-specific Bt maize, the response was also negative when all maize Cry proteins were pooled. Based on these general results, we examined all non-target effects by pooling all events within each of cotton and maize to maximize sample sizes for each comparison. Only a single construct (Cry3A) was present for potatoes.

### Bt crop vs. non-transgenic control (no insecticides used)

#### Functional guild analyses

**Figure 1:**
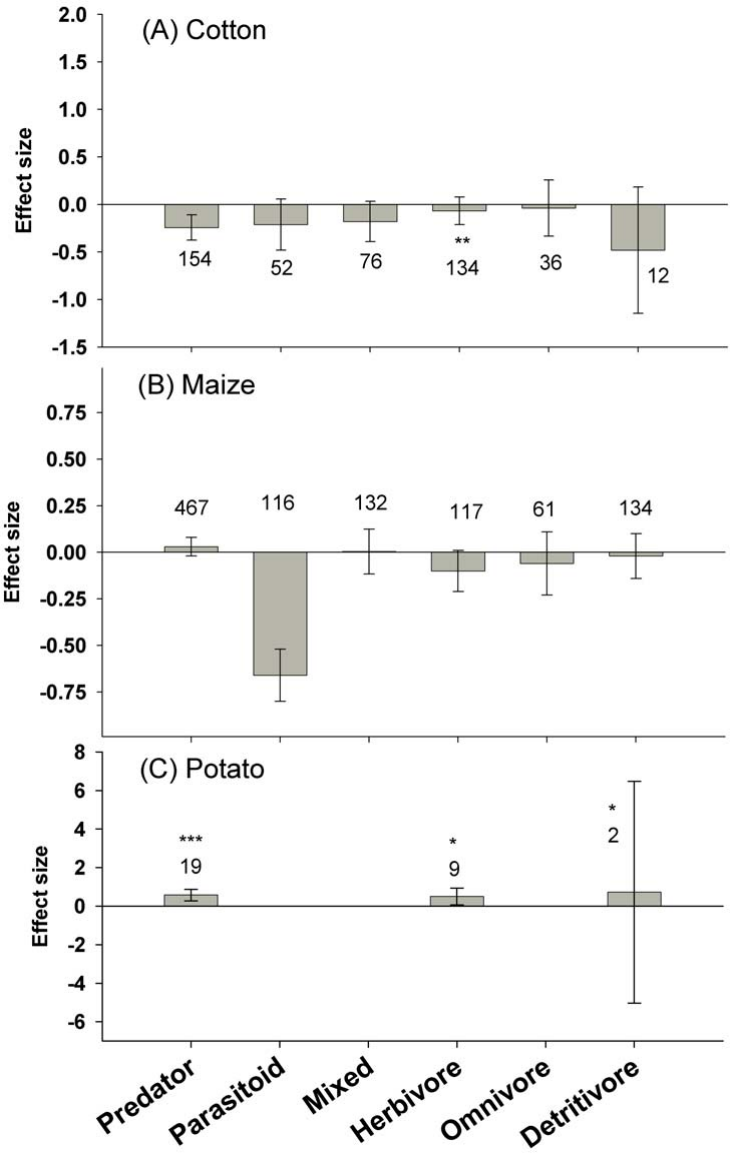
The effect of Bt crops on non-target functional guilds compared to unsprayed, non-Bt control fields. Bars denote the 95% confidence intervals, asterisks denote significant heterogeneity in the observed effect sizes among the comparisons (* <0.05, ** <0.01, *** <0.001), and Arabic numbers indicate the number of observations included for each functional group.

In cotton, there were slightly fewer predators in Bt cotton fields compared to unsprayed, non-transgenic fields (E = −0.24±0.13, n = 154, Fig. 1a). This result was not related to feeding style within this functional group but was largely driven by the lower abundance of Nabidae and Coccinellidae (E = −0.68±0.31 n = 31) in Bt fields. Removal of these families caused the effect size for predators to become non-significant. Effect sizes varied within the herbivore guild (significant heterogeneity) but further partitioning by feeding style or by order and family revealed no differences in abundance with the limited sample sizes available for these analyses (Thysanoptera, n = 4; Lepidoptera, n = 15; Acarina, n = 10; Diptera, n = 6). The abundance of common predatory genera, including *Chrysoperla* (n = 5), *Orius* (n = 9), *Geocoris* (n = 15) and of two species associated with predicted or documented nontarget pest outbreaks in Bt cotton (*Lygus* spp, n = 7; *Bemisia tabaci* n = 13) were similar in Bt and non-Bt cotton.

In maize, analyses revealed a large reduction of parasitoids in Bt fields (E = −0.72±0.14, n = 116, Fig. 1b). This effect stemmed from the lepidopteran-specific maize hybrids, and examining the 116 observations showed that most (n = 93) were conducted on *Macrocentrus grandii*, a specialist parasitoid of the Bt-target, *Ostrinia nubilalis*. There was no significant effect on other parasitoids (E = −0.02±0.33, n = 23), but *M. grandii* abundance was severely reduced by Bt maize (E = −0.84±0.16, n = 93). Higher numbers of the generalist predator, *Coleomegilla maculata*, were associated with Bt maize (E = 0.14±0.13; n = 37) but numbers of other common predatory genera (*Orius*, n = 81; *Geocoris*, n = 4, *Hippodamia*, n = 18; *Chrysoperla*, n = 32) were similar in Bt and non-Bt maize.

For potatoes, there were more predators (E = 0.58±0.30, n = 19) and herbivores (E = 0.50±0.43, n = 10) in Bt potato fields than in unsprayed control fields (Fig. 1c). Significant heterogeneity existed in both of these functional groups, but sample sizes did not allow finer analyses.

#### Predator-non target herbivore ratio analyses

We found no evidence for changes in the ratio of predators to non-target herbivores in any Bt crop. Data for this analysis were available from 12, 25 and 2 experiments in cotton, maize and potato, respectively.

#### Predator-detritivore analyses

Most of the detritivores in the database were Collembola, and carabid and staphylinid beetles were their primary predators. We found no significant effects of Bt crops on detritivores overall, on any of the five collembolan families or their carabid and staphylinid beetle predators, or on the non-collembolan detritivore families Lathridiidae and Japygidae.

### Unsprayed Bt crop vs. non-transgenic control sprayed with insecticide

#### Functional guild analyses

**Figure 2:**
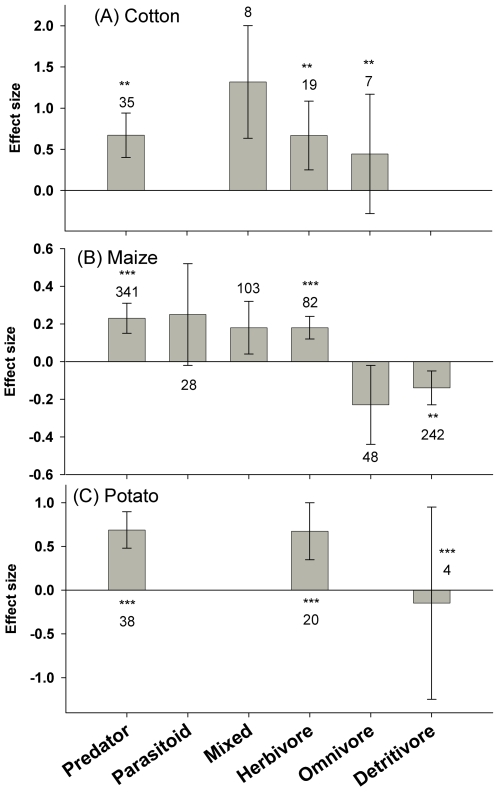
The effect of Bt crops on non-target functional guilds compared to insecticide-treated, non-Bt control fields. Bars denote the 95% confidence intervals, asterisks denote significant heterogeneity in the observed effect sizes among the studies (* <0.05, ** <0.01, *** <0.001), and Arabic numbers indicate the number of observations included for each functional group.

In cotton, there were many more predators, herbivores and mixed-guild taxa in unsprayed Bt cotton fields than in insecticide sprayed controls (Fig. 2a). For common predator species, there were more *Geocoris* spp. (E = 2.0±1.5; n = 4) in Bt cotton but no detectable difference in *Orius* spp. (n = 2) or *Chrysoperla* spp. (n = 2) for the limited studies available. There was a greater abundance of the non-target pest species *B. tabaci* (E = 1.6±1.3, n = 4) in Bt cotton fields compared to a non-transgenic control sprayed with insecticide, but no detectable change in abundance with the small number of studies on *Lygus* spp. (n = 2).

There was significant heterogeneity within effect sizes for predators, herbivores and omnivores. Overall sample sizes were limited but analyses by feeding style or taxonomic order within these functional groups revealed highly variable responses. Predator effect sizes were consistently positive but varied in magnitude from 0.2 (Neuroptera) to 1.2 (Diptera). Consistently positive effect sizes were also observed for Hemipteran herbivores and ranged from 0.9 (Miridae) to 3.2 (Cicadellidae). For other functional guilds, none of the effect sizes for orders or family were significant. In contrast to maize (see below), the use of pyrethroid insecticides in control fields did not influence effect sizes nor explain overall heterogeneity.

**Figure 3:**
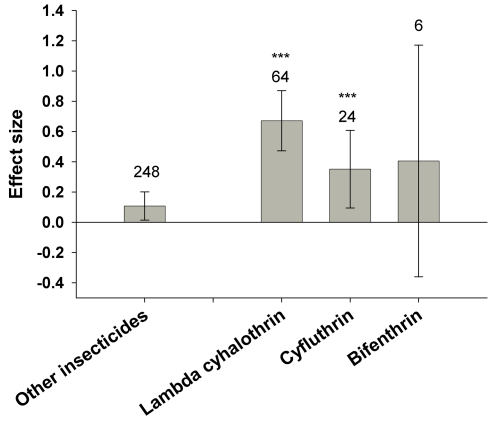
Effects of Bt maize vs. control fields treated with a pyrethroid insecticide on predatory arthropods. Bars denote the 95% confidence intervals, asterisks denote significant heterogeneity in the observed effect sizes among the studies (* <0.05, ** <0.01, *** <0.001), and Arabic numbers indicate the number of observations included for each functional group.

In maize, the abundance of predators (E = 0.23±0.08, n = 341) and members of the mixed functional guild (E = 0.18±0.14, n = 103) were higher in Bt maize compared to insecticide-sprayed controls (Fig. 2b). Significant heterogeneity occurred in predators, indicating variation in the effects of Bt maize on this guild. For example, we detected no significant effect sizes for the common predator genera *Coleomegilla* (n = 20), *Hippodamia* (n = 7) or *Chrysoperla* (n = 13), but the predator *Orius* spp. and the parasitoid *Macrocentrus* were more abundant in Bt maize than in non-Bt maize plots treated with insecticides (E = 0.38±0.17; n = 75; E = 0.48±38, n = 9, respectively). Partitioning by taxonomic groupings or the target toxin (Lepidoptera versus Coleoptera) did not reduce heterogeneity within predators. However, insecticides differentially affected predator populations. Specifically, application of the pyrethroid insecticides lambda-cyhalothrin, cyfluthrin, and bifenthrin in non-Bt control fields resulted in comparatively fewer predators within these treated control plots. Omitting studies involving these pyrethroids revealed a much smaller and homogeneous effect size (E = 0.11±0.095, n = 248). Predator abundance in Bt fields was still significantly higher compared with insecticide-treated plots, but the difference was less marked without the pyrethroids (Fig. 3). Compared to the subset of controls using pyrethroids, Bt maize was particularly favorable to *Orius* spp. (E = 1.67±0.66; n = 9), and Araneae (0.73±0.27; n = 32).

In contrast to the positive effects on most functional guilds, there were fewer omnivores (E = 0.23±0.21, n = 45) and detritivores (E = −0.14±0.09, n = 242) in Bt maize than in insecticide-treated controls. The decline in omnivores was completely explained by effects on Formicidae (E = −0.43±0.31, n = 24) and removal of this group led to an insignificant effect size (E = −0.05±0.33, n = 21). Significant heterogeneity existed in effect sizes for the detritivore category. One factor that explained some of the variability is that the pyrethroid, cyfluthrin, had little effect on the detritivores, whereas the other pyrethroids were detrimental to this group. Eliminating the cyfluthrin studies (n = 6) was sufficient to eliminate the heterogeneity (*P* = 0.17, n = 236, E = 0.17±0.09).

Bt-maize favored non-target herbivore populations relative to insecticide-treated controls, but there was also significant heterogeneity, some of which was explained by taxonomy. Aphididae were more abundant in insecticide sprayed fields (E = −0.42±0.28, n = 25) and Cicadellidae occurred in higher abundance in the Bt maize (E = 0.77±0.27, n = 22). In contrast to patterns associated with predators and detritivores, type of insecticide did not explain the heterogeneity in herbivore responses. The pyrethroid-treated controls accounted for 85% of the herbivore records. Individual pyrethroids had variable effects on this group, and none yielded strong effects on the herbivores. An underlying factor associated with the heterogeneity of the herbivore guild remained unidentified, but many possible factors were eliminated (e.g., Cry protein target, Cry protein, event, plot size, study duration, pesticide class, mechanism of pesticide delivery, sample method, and sample frequency).

The “mixed” functional group was more abundant in Bt maize (E = 0.18±0.14, n = 103) compared with non-*Bt* maize treated with insecticides. The majority of this functional group is comprised of carabids (n = 33), nitidulids (n = 26), and mites (n = 23).

For potatoes, the abundance of predators (E = 0.69±0.30, n = 38), but not herbivores, was significantly higher in the Bt crop (Fig 2c). Responses within each functional group were variable but sample sizes were too low to further partition this significant heterogeneity.

#### Predator-non target herbivore ratio analyses

No significant change in predator-prey ratios was detected in cotton or potato; in maize there was a significantly higher predator- prey ratio in *Bt* maize plots than in the insecticide controls (E = 0.63±0.42, n = 15). Significant heterogeneity for the predator: prey response existed in all three crops, but again sample sizes were too small to explore the cause of this variability.

#### Predator-detritivore analyses

**Figure 4:**
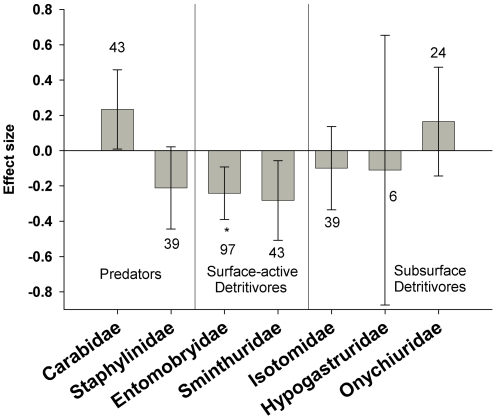
Effect of Bt crops vs. insecticide-treated, non-Bt control fields on soil-inhabiting predators and detritivores. Bars denote the 95% confidence intervals, asterisks denote significant heterogeneity in the observed effect sizes among the studies (* <0.05, ** <0.01, *** <0.001), and Arabic numbers indicate the number of observations included for each functional group.

The higher abundance of detritivores in sprayed non-Bt maize appeared to be driven primarily by two families of Collembola with a high proportion of surface-active species (Entomobryidae: E = −0.24±0.15, n = 97; Sminthuridae: E = −0.28±0.23, n = 43, Fig. 4). Three other families, Isotomidae, Hypogastruridae, and Onychiuridae, with more sub-surface species, were similar in Bt and non-Bt fields. We would expect surface-active collembolans to be more vulnerable to surface-active predators, and we detected a significantly lower abundance in one predator of Collembola (Carabidae: E = 0.23±0.22, n = 43) but not in another (Staphylinidae: E = −0.21±0.23, n = 39, Fig 4). The other two detritivore families occupy different niches than Collembola and responded differently to insecticide treatments. The abundance of Japygidae (Diplura) was unchanged (E = −0.11±0.35, n = 9), but that for Lathridiidae (Coleoptera) was higher in Bt maize (E = 0.76±0.70, n = 6), suggesting a direct negative effect of insecticides on this latter group. Lathridiid beetles, although being surface-active humus-feeders, are larger and more motile than Collembola and thus may be less vulnerable to predators and more vulnerable to insecticides.

### Bt crop sprayed with insecticide vs. non-transgenic control sprayed with insecticide

#### Functional guild analyses

**Figure 5:**
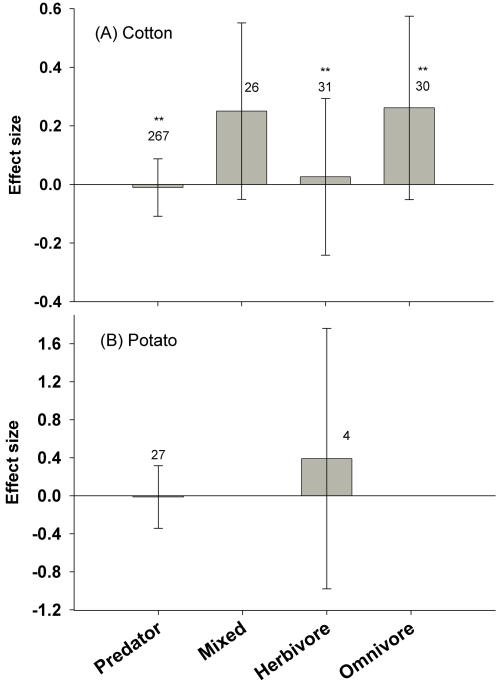
Effect of insecticide-treated Bt crops vs. insecticide-treated non-Bt control field on non-target functional guilds. Bars denote the 95% confidence intervals, asterisks denote significant heterogeneity in the observed effect sizes among the studies (* <0.05, ** <0.01, *** <0.001), and Arabic numbers indicate the number of observations included for each functional group.

In cotton, the abundance of all functional guilds was similar between Bt and non-Bt fields (Fig. 5a). No studies were available on parasitoids. The abundances of common predator genera *Geocoris* (n = 45), *Orius* (n = 18) and *Chrysoperla* (n = 12) or the pest *Lygus* spp. (n = 20) were similar between crops. No studies were available for *B. tabaci*. Significant heterogeneity existed within the predator, herbivore and omnivore functional guilds. Further analyses by feeding style and taxonomic level revealed variable but consistently nonsignificant effect sizes within these guilds. No studies using maize were available for this comparison. In potatoes, studies available measured effects on only two functional groups: predators (n = 27) and herbivores (n = 4), and neither effect was significant (Fig. 5b).

#### Predator-non-target herbivore ratio analyses

Studies for predator-prey analysis were only available for cotton (n = 12) and potato (n = 2), and we detected no change in predator-prey ratios.

## Discussion

When comparing Bt plants to their non-transgenic counterparts without any additional insecticides, meta-analysis revealed no uniform negative or positive effects on ecological functional guilds. Predators were slightly lower in abundance in Bt cotton but no other effects were detected for other functional guilds in this crop. This negative effect on predators was not seen in the two other cropping systems; in fact, this guild was favored by Bt potato. The small negative effect on predators as a group was not driven by any common individual species that we analyzed but rather by more moderate reductions in two predaceous families (Nabidae and Coccinellidae), a pattern identified in several non-target studies [35], [36]. We detected no change in the abundance of aphids as a group, a common prey item for coccinellids, so common prey reduction probably does not explain the decrease of these predators. Reductions in target prey could be a contributing factor, especially for nabids [35], [36]; however, other explanations, such as sublethal effects of feeding on Bt pollen or other prey abundance or quality issues in Bt fields cannot be eliminated for either group [35]. We detected no significant effect size on predators as a group in maize; however, studies indicate a higher abundance of one common predator genus, *Coleomegilla*, in Bt fields compared to unsprayed non-Bt fields. Therefore, we identified a species-specific effect in Bt maize but no consistent effects on any of the functional guilds.

Our analysis corroborates the strong negative effect of Bt maize on specialist parasitoids reported in the literature [15]. However, a closer examination suggests that most of the parasitoid studies in this system focus on the abundance of *M. grandii*, which specializes on the target pest. From the limited number of studies on other parasitoids, there was no detectable effect on parasitoids; however, more studies will be needed to resolve whether there is a general effect on parasitoids (Appendix S3).

In general, we also found no changes in select individual genera or species that have been the subject of some debate in the literature. For example, several studies [37], [38] have documented greater abundance of mirid bugs (e.g., *Lygus* spp.) in Bt cotton fields. Ponsard et al. [16] noted a moderate reduction in longevity of *Geocoris punctipes* and *Orius tristicolor* when these predators fed on Cry1Ac intoxicated prey in the laboratory, and Gutierrez et al. [39] predicted that due to this effect we might see increased abundance and pest status in *Lygus* spp. and *B. tabaci* in Bt cotton. Our meta-analyses showed no change in the abundance of *Geocoris* spp., *Orius* spp., *Lygus* spp. or *B. tabaci* in Bt cotton over multiple studies, even though ingestion of Bt toxins have been confirmed for the two predator genera [40], [41]. Perhaps most significantly, our analyses consistently failed to detect any changes in the abundance of *Chrysoperla* spp. in Bt cotton or Bt maize. This group has been the subject of intense debate in the literature (e.g., [42]–[44]). While the conclusions from any individual analysis should be viewed cautiously because sample sizes were small (range = two to 32), collectively our analysis would suggest that even if small changes in life history parameters are altered by Bt toxins, they are not reflected in altered levels of field abundance in Bt crops.

Generally speaking, Bt crops favored the abundance of non-target arthropods relative to insecticide-treated controls, especially within the predator, mixed, and herbivore functional guilds. Insecticide-treated fields consistently had fewer predators than Bt fields, but the degree to which predators decreased varied among the different insecticides applied to the non-Bt fields. This is well illustrated in the maize system, where three pyrethroid insecticides were particularly deleterious to certain predators, namely *Orius insidiosus* and spiders. In the U.S. pyrethroids such as lambda-cyhalothrin, bifenthrin, and cyfluthrin, are used widely in sweet corn production but not field corn production [45]. These insecticides are used on a small percentage of field corn acreage; in 2005 surveys, cyfluthrin was used on 7% of acreage (∼76.5 million acres), bifenthrin on 2% and lambda cyhalothrin on 1% [46]. Because foliar-applied insecticides are infrequently used in field-corn production, the adoption of lepidopteran-specific Bt-maize probably has had little effect, *pro* or *con*, on predator populations.

In cotton, the magnitude of the positive effect on predators, compared to insecticide-sprayed fields, was variable within the guild, but was not explained by taxonomy, feeding style or choice of insecticide in the treated control. Limited sample sizes for individual species comparisons existed but indicated a strong positive effect on the common predator *Geocoris* spp. Therefore, the larger number of predators in the unsprayed Bt cotton fields was in part probably due to a higher abundance of a common species. Although herbivore abundance did not differ between Bt and sprayed non-Bt cotton fields, we did detect higher abundance of one nontarget pest, *B. tabaci,* in Bt cotton fields. This is consistent with the use of insecticides for managing this pest in non-Bt cotton control fields. Similarly, the higher abundance of Aphididae in insecticide sprayed maize fields, compared to unsprayed Bt maize, reflect the expected resurgence in a pest population following insecticide treatment [47].

Two groups that were more abundant in insecticide treated controls within the maize system were omnivores and detritivores. Many of the arthropods in these systems are soil dwelling, and may have had reduced exposure to foliar-applied insecticides. Still, if this were the sole factor affecting their abundance, we might expect to see the same pattern in the comparison with the untreated controls. It seems likely that the insecticides are disrupting the community dynamics to produce the observed increase in abundance of omnivores and detritivores. The generally positive effects of insecticides on the surface-active Collembola are probably indirect and may result from a corresponding decrease in the abundance of their predators, which include carabid and staphylinid beetles [34]. Some subsurface species that are rarely preyed upon by surface-active predators showed the opposite response of being directly reduced by soil insecticides. Finally, the differing responses of other non-collembolan detritivores suggest that insecticides might affect their populations through means other than the predator-prey interactions, although data on these groups were limited. For example, lathridiid beetles are larger and more motile surface-active humus feeders [27] than springtails and may be less vulnerable to predators, such as small staphylinids or carabids, and more vulnerable to insecticides. We conclude that Bt crops altered the interaction between certain detritivores and their predators to a much smaller extent than did insecticide applications.

In cotton, similar abundances of non-target functional guilds occurred when both Bt and control fields were sprayed with insecticides. There were no data available in the database on insecticides used in these studies, but it is likely that different insecticides would be used in the Bt and non-Bt when both are sprayed because the pest complexes would be altered by the use of Bt crops. The strength of these experiments is that the studies represent farmer use of Bt cotton because they were largely conducted on commercial farms; therefore, they reflect the net effect of Bt cotton in a system with multiple pest species, many of which are not susceptible to Bt toxins [48]. From the meta-analysis, the net effect of “commercial practice” for the locations studied indicates an equivalency of Bt and non-Bt systems with respect to functional guilds of non-target arthropods.

Limited evidence suggested that changes in species interactions occurred not due to the use of Bt crops, but rather due to the use of insecticides. Higher predator to herbivore ratios occurred in Bt maize than in the insecticide-treated non-Bt control. We found no other evidence of effects on species interactions between predators and prey; however, only a limited number of studies were available for the analysis. Lack of effect in sprayed treatments (e.g. cotton) would suggest that the abundances of both herbivores and predators were affected equally by insecticide use so that the ratio does not change compared with an untreated situation. These meta-analysis results based on synthesizing data on all predators and herbivores within multiple studies are consistent with the findings of predator to prey ratios calculated for two insect pests in the cotton system [21].

As more data accumulate on the ecological impacts of Bt crops or other GE organisms, syntheses like meta-analyses will continue to be a useful tool to guide the decisions of diverse stakeholders such as developers, regulators, researchers, growers and consumers. With emphasis on guilds, our analyses focused on ecological function rather than on biodiversity or taxon-based abundance [49] and provide for a more generalized assessment of impact of Bt crops within agricultural systems. For example, our results suggest that while functional guilds should continue to be the focus of tiered-testing systems in terms of species selection, the relatively consistent effects of Bt crops on members within a guild indicate that some latitude exists to choose species with more desirable traits for laboratory culture and handling without sacrificing realism when assessing impacts within agriculture. The relatively consistent responses within guilds would also argue for greater emphasis on representation by functional role rather than taxonomic affinity in field studies. This would perhaps allow more targeted and accurate sampling methods to be developed for fewer taxa representing functional guilds while improving statistical power and overall robustness in non-target assessment. Furthermore, additional analyses with the “Bt crop effects on non-targets” database [13] could provide additional insights into how Bt effects detected under laboratory testing reflect the trends and effects that Marvier et al. [13] and we have detected using field studies.

Our synthesis of available studies also reveals where research efforts have been intensive and where gaps exist. For example, studies have consistently documented impacts on the parasitoid *M. grandii*, but relatively few field studies have been conducted on other parasitoids. Choosing other species that account for larger parasitism rates in agricultural systems will improve our understanding of how Bt crops affect parasitoids and whether any effects are due to the direct effect of Bt toxin or to prey in poor condition from feeding on Bt toxin. Our experience with the database also points to the need for more consistent reporting of data in the literature. In order to include studies in meta-analyses that require measures of error, publications need to report sample sizes and standard errors (or standard deviations). A large number of comparisons in the database compiled by Marvier et al. [13] do not include these error terms. Specifically, we were unable to include 567 observations of 123 experiments from 43 different field studies. Inclusion of these studies would have increased the sample sizes for every analysis between 10 and 140%.

Debate over issues of food and environmental safety, regulatory oversight, and welfare of the farming community as a whole are likely to continue as GE technology moves forward with new crops, new traits and new adopting countries. Our meta-analysis reinforces the notion that Bt crops are one tool in the integrated pest management toolbox and that their comparative environmental impact on non-target organisms will depend on how these tools are integrated and applied within agricultural production systems.

### Limitations

A number of reviews have provided qualitative syntheses of the impact of Bt crops on non-target organisms [7], [9]–[11], [50], [51] but more detailed, quantitative syntheses are lacking. Meta-analysis provides one such quantitative approach and has a long history in many fields of study including ecology over the last 15 years [52]–[54]. Meta-analysis is not without its limitations [55], but we paid careful attention here to the critical issues of non-independence, found no evidence of publication bias [53], revealed no consistent and meaningful effects of experimental design variables, and discovered a lack of sensitivity to the particular studies included in our database, especially for cotton and maize.

The examination of non-target and other ecological effects from Bt and other GE crops is a quickly growing field of investigation with new studies being published at a rapid rate. Although there have been several dozen new non-target studies published since the database was compiled, cumulative meta-analysis indicates a convergence of the results presented here (Appendix S3). Therefore, adding very recent studies is unlikely to alter our conclusions on the effects of Bt crops on ecological function within agroecosystems for the events and Bt toxins examined.

## Supporting Information

Appendix S1 Dataset used for analyses
(4.46 MB XLS)

Appendix S2 Taxonomic groups associated with functional guilds.
(0.16 MB DOC)

Appendix S3 Cumulative meta-analyses for effects on functional guilds
(0.44 MB DOC)
