# Supplementary material for: Bt Crop Effects on Functional Guilds of Non-Target Arthropods: A Meta-Analysis
Source: PLoS One. 2008 May 7;3(5):e2118. doi: 10.1371/journal.pone.0002118 (PMC2346550; doi:10.1371/journal.pone.0002118)
Supplement: Appendix S2 — Taxonomic groups associated with functional guilds. (0.16 MB DOC) [file pone.0002118.s002.doc]

| Phylum | Class | Order | Family | *Genus* |
| --- | --- | --- | --- | --- |
| **Detritivores** |  |  |  |  |
| Annelida | Oligochaeta^1^ |  |  |  |
| Arthropoda | Entognatha | Diplura | Japygidae^1^ |  |
|  | Insecta | Coleoptera | Languriidae^1^ |  |
|  |  |  | Lathridiidae^1^ | *Cortinicara* |
|  |  | Collembola^1^ | Entomobryidae^1^ | *Entomobrya, Lepidocyrtus, Orchesella, Pseudosinella, Sinella, Tomocerus* |
|  |  |  | Hypogastruridae^1^ | *Hypogastrura* |
|  |  |  | Isotomidae^1^ | *Folsomides, Isotoma, Isotomodes,* |
|  |  |  | Onychiuridae^1^ | *Onychiurus, Tullbergia* |
|  |  |  | Sminthuridae^1^ | *Bourletiella, Sminthurinus, Sphaeridia* |
|  |  | Diptera | Drosophilidae^1^, Heleomyzidae^1^, Lauxaniidae^1^, Muscidae^1^, Scatopsidae^1^, Sciaridae^1^, Tipulidae^1^ |  |
|  |  | Psocoptera^1^ |  |  |
| **Herbivores** |  |  |  |  |
| Arthropoda | Arachnida | Acarina^1^ |  |  |
|  | Insecta | Coleoptera | Cerambycidae^1^, Cetoniidae^1^, Curculionidae^1^, Scarabaeidae^1^, Tenebrionidae^1^ |  |
|  |  |  | Chrysomelidae^1^ | *Altica, Chaetocnema, Oulema* |
|  |  | Diptera | Agromyzidae^1^, Cecidomyiidae^1^, Fergusoninidae^1^, Tephritidae^1^ |  |
|  |  |  | Otitidae | *Euxesta* |
|  |  | Hemiptera | Aleyrodidae^1^ | *Bemisia* |
|  |  |  | Alydidae^1^ |  |
|  |  |  | Aphididae^1^ | *Macrosiphum, Metopolophium, Rhopalosiphum, Sitobion,* |
|  |  |  | Aphidoidea^1^, Auchenorrhyncha^1^ |  |
|  |  |  | Cicadellidae^1^ | *Empoasca, Macrosteles, Psammotettix, Zyginidia* |
|  |  |  | Delphacidae | *Laodelphax, Nilaparvata, Sogatella* |
|  |  |  | Lygaeidae^1^ | *Nysius, Oxycarenu* |
|  |  |  | Miridae^1^ | *Lygus, Pseudatomoscelis, Taylorilygus* |
|  |  |  | Pentatomidae | *Acrosternum, Nezara* |
|  |  |  | Pyrrhocoridae | *Dysdercus* |
|  |  |  | Scutelleridae | *Tectocoris* |
|  |  |  | Sternorrhyncha^1^ |  |
|  |  | Hymenoptera | Agaonidae^1^ |  |
|  |  | Lepidoptera^1^ | Gelechiidae | *Phthorimaea* |
|  |  |  | Geometridae^1^ |  |
|  |  |  | Noctuidae | *Pseudoplusia, Sesamia, Spodoptera* |
|  |  |  | Tortricidae | *Crocidosema* |
|  |  | Orthoptera | Acrididae^1^ |  |
|  |  | Thysanoptera | Phloeothripidae | *Haplothrips* |
|  |  |  | Thripidae^1^ | *Chirothrips, Frankliniella, Limothrips* |
| **Mixed** |  |  |  |  |
| Arthropoda | Arachnida | Acarina | Astigmata^1^, Oribatei^1^, Prostigmata^1^ |  |
|  | Insecta | Coleoptera^1^ | Anthicidae, Cetoniidae, Curculionidae, Lathridiidae, Scarabaeidae, Carabidae, Cucujidae, Nitidulidae, Phalacridae |  |
|  |  | Coleoptera, Diptera, Neuroptera^2^ | Chrysopidae, Coccinellidae, Hemerobidae, Syrphidae |  |
|  |  | Dermaptera^1^ |  |  |
|  |  | Diptera^1^ | Brachycera, Chloropidae, Ephydridae, Phoridae, Platystomatidae, Psychodidae |  |
|  |  | Hemiptera^1^ | Pentatomidae |  |
|  |  | Hymenoptera^1^, Orthoptera^1^, Thysanoptera^1^ |  |  |
| **Omnivores** |  |  |  |  |
| Arthropoda | Insecta | Blattodea^1^ |  |  |
|  |  | Coleoptera | Carabidae | *Calathus, Dolichus, Harpalus* |
|  |  |  | Elateridae^1^*,* Mycetophagidae^1^ |  |
|  |  |  | Nitidulidae | *Carpophilus* |
|  |  | Dermaptera | Carcinophoridae | *Euborellia* |
|  |  |  | Forficulidae | *Forticula* |
|  |  |  | Labiduridae^1^ | *Labidura* |
|  |  | Diptera | Anthomyiidae^1^*,* Ceratopogonidae^1^ |  |
|  |  |  | Chironomidae^1^ | *Smittia* |
|  |  | Hemiptera | Miridae | *Campylomma, Rhinacloa, Spanogonicus* |
|  |  | Hymenoptera | Formicidae^1^ | *Iridomyrmex, Rhytidoponera, Solenopsis* |
|  |  | Orthoptera | Gryllidae^1^ |  |
| **Parasitoids** |  |  |  |  |
| Arthropoda | Insecta | Diptera | Pipunculidae^1^*,* Pyrgotidae^1^ |  |
|  |  |  | Tachinidae^1^ | *Lydella, Pseudoperichaeta* |
|  |  | Hymenoptera | Aphelinidae^1^, Bethylidae^1^ , Ceraphronidae^1^, Chalcididae^1^, Chalcidoidea^1^, Diapriidae^1^, Elasmidae^1^, Encyrtidae^1^, Eucoilidae^1^, Eupelmidae^1^, Figitidae^1^, Mymaridae^1^, Mymarommatidae^1^, Perilampidae^1^, Platygastridae^1^, Pteromalidae^1^, Torymidae^1^, Trichogrammatidae^1^ |  |
|  |  |  |  |  |
|  |  |  | Braconidae^1^ | *Macrocentrus* |
|  |  |  | Eulophidae^1^ | *Hemiptarsenus* |
|  |  |  | Ichneumonidae | *Eriborus* |
|  |  |  | Scelionidae^1^ | *Telenomus* |
| **Predators** |  |  |  |  |
| Arthropoda | Arachnida | Acarina | Mesostigmata^1^ |  |
|  |  | Araneae^1^ | Clubionidae^1^ Oxyopidae^1^, Salticidae^1^, Theridiidae^1^ |  |
|  |  |  | Dictynidae | *Dictyna* |
|  |  |  | Thomisidae^1^ | *Misumenops,* |
|  | Chilopoda^1^ |  |  |  |
|  |  | Coleoptera | Anthicidae | *Notoxus* |
|  |  |  | Cantharidae^1^, Cleridae^1^, Histeridae^1^, Staphylinidae^1^ |  |
|  |  |  | Carabidae | *Lebia, Microlestes, Poecilus, Scarites, Trechus* |
|  |  |  | Cicindelidae^1^ | *Cicindela, Megacephala* |
|  |  |  | Coccinellidae^1^ | *Adalia, Coccinella, Coleomegilla, Cycloneda, Diomus, Microweisea, Harmonia, Hippodamia, Propylea, Psyllobora, Scymnus* |
|  |  |  | Melyridae^1^ | *Collops, Dicranolaius* |
|  |  | Dermaptera | Forficulidae | *Doru* |
|  |  | Diptera | Dolichopodidae^1^ |  |
|  |  |  | Empididae^1^ | *Drapetis* |
|  |  |  | Syrphidae^1^ | *Syrphus* |
|  |  | Hemiptera | Anthocoridae^1^ | *Orius* |
|  |  |  | Geocoridae | *Geocoris* |
|  |  |  | Miridae | *Deraeocoris* |
|  |  |  | Nabidae^1^ | *Nabis, Nabus* |
|  |  |  | Pentatomidae | *Cermatulus, Oechalia, Podisus, Stiretrus* |
|  |  |  | Reduviidae^1^ | *Sinea, Zelus* |
|  |  | Neuroptera^1^ | Chrysopidae^1^ | *Chrysoperla* |
|  |  |  | Hemerobiidae^1^ | *Hemerobius, Micromus* |
|  |  | Odonata^1^ |  |  |

^1^Indicates that some studies specified only this level of taxonomy,

^2^Used as a taxonomic grouping in some studies
