# Supplementary material for: Bt Crop Effects on Functional Guilds of Non-Target Arthropods: A Meta-Analysis
Source: PLoS One. 2008 May 7;3(5):e2118. doi: 10.1371/journal.pone.0002118 (PMC2346550; doi:10.1371/journal.pone.0002118)
Supplement: Appendix S3 — Cumulative meta-analyses for effects on functional guilds (0.44 MB DOC) [file pone.0002118.s003.doc]

## **Appendix S3. Cumulative meta-analyses of effects on functional guilds**

Cumulative meta-analysis is a means of examining the influence of successive studies on effect size as they are added to the database and can provide a measure of convergence in the dataset [1]. Non-target studies in Bt and other GE crops continue to be published at a rapid rate. Cumulative analyses can reveal broader trends in study data that may allow more definitive conclusions to be drawn despite the addition of new information [2]. Our cumulative meta-analysis for each functional guild pooled over all three crops shows the mean effect size and its 95% confidence interval as study observations are accrued over time. The consistency of the mean effect size and the narrowing of the confidence interval with increasing study observations indicate a convergence over time to a particular effect size (Figs. S3-1, S3-2). The plots demonstrate convergence in effect sizes that are generally consistent with our crop-specific results. The pooling of crops did indicate one difference from separate analyses. For maize we report no significant effect of Bt crops on parasitoids other than *Macrocentrus* in comparison to non-Bt controls, but the cumulative meta-analysis suggests that studies may be converging on a negative effect size (Fig. S3-1). Thus, future studies will likely be needed to resolve the effects of Bt crops in general on parasitoids other than those specializing on target pests. Analyses further re-emphasize the small sample size for parasitoids when comparing Bt crops to insecticide-treated non-Bt controls (Fig. S3-2).

## **References**

1. Rosenberg MS, Adams DC, Gurevitch J (2000) MetaWin version 2: statistical software for meta-analysis. Sunderland, Massachusetts, USA: Sinauer Associates, Inc.

2. Lau, J, Antman, EM, Jimenez-Silva, J, Kupelnick, B, Mosteller, F, Chalmers, TC. (1992) Cumulative meta-analysis of therapeutic trials for myocardial infarction. New England Journal of Medicine 327: 248-254.


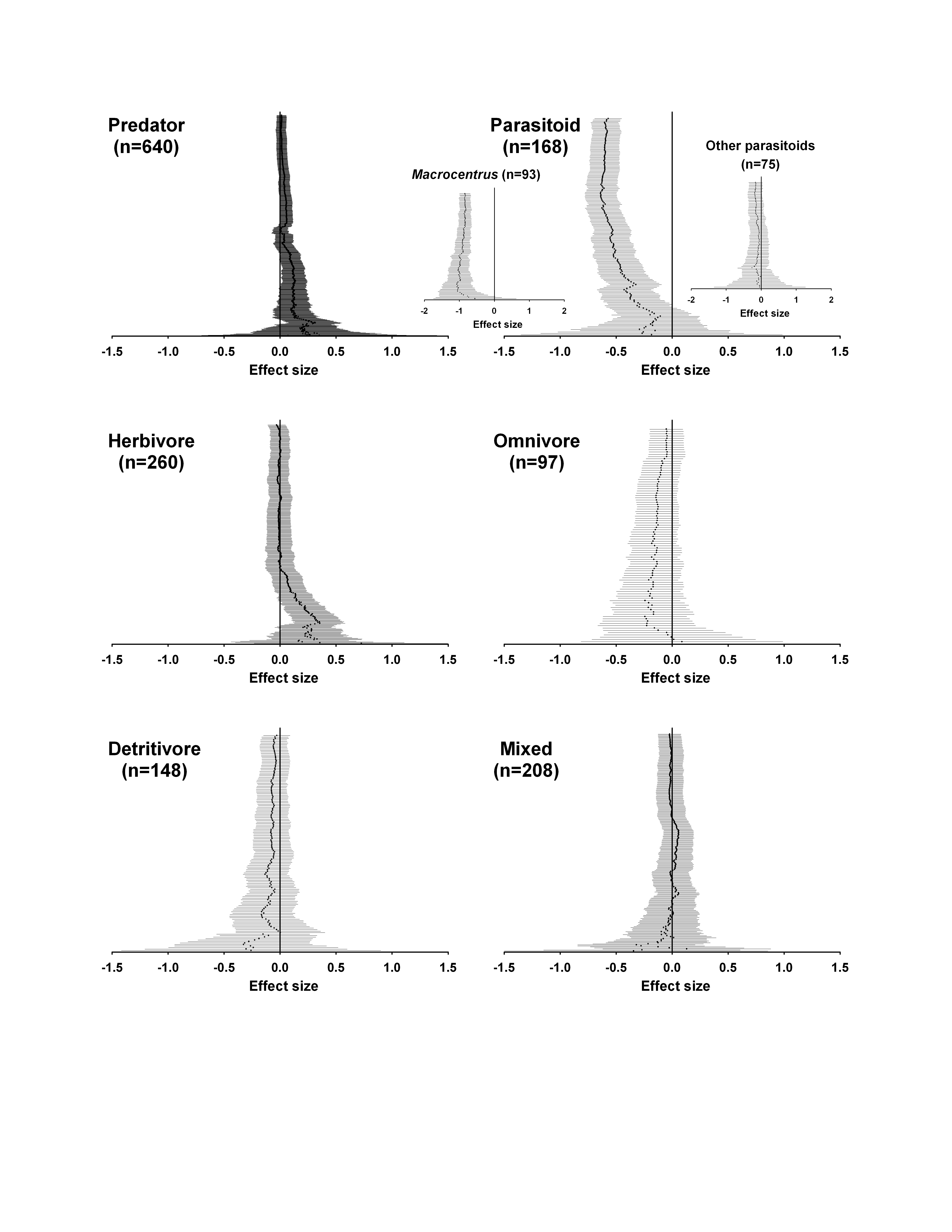


Fig. S3-1. Cumulative effect size of all Bt crops pooled when compared to non-Bt controls receiving no insecticides.


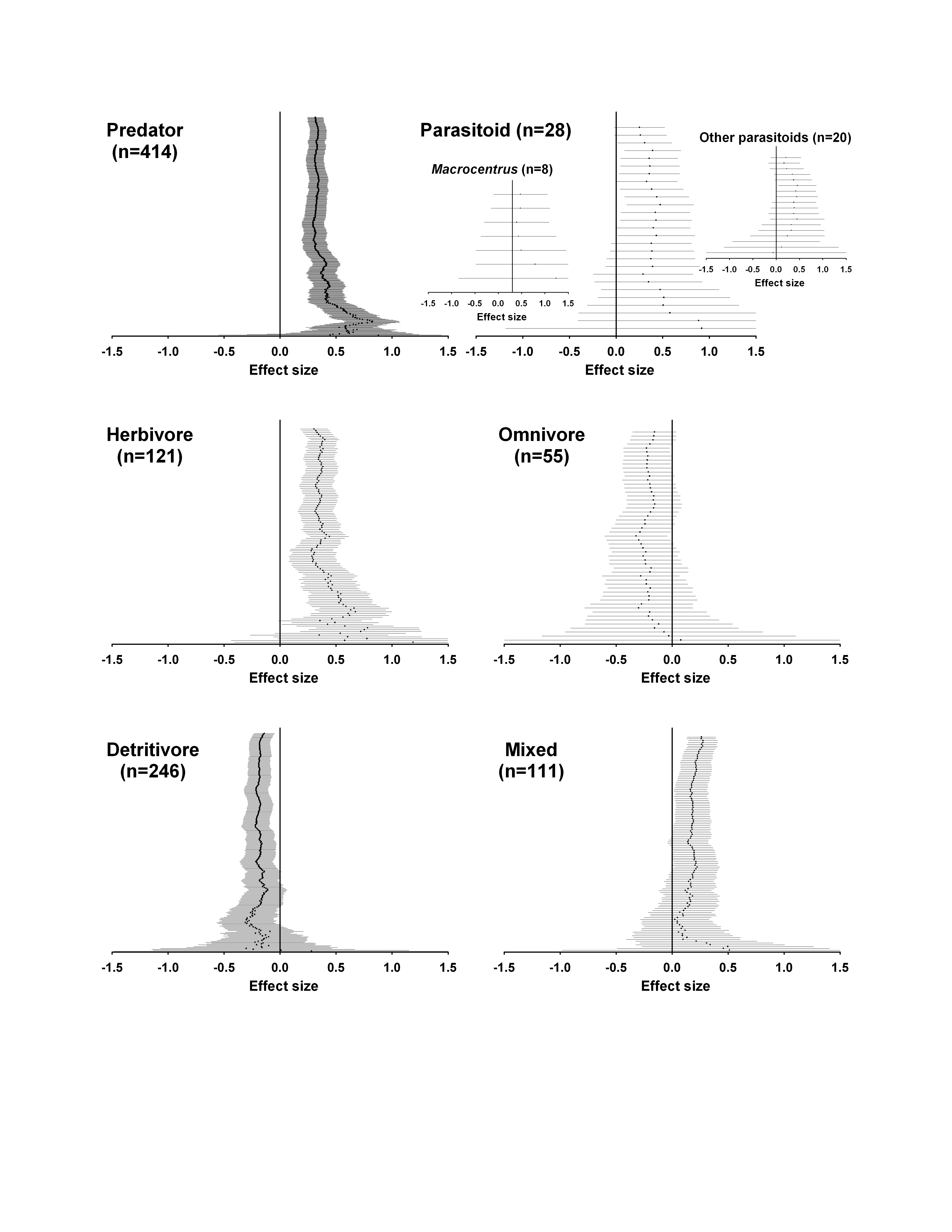


Fig. S3-2. Cumulative effect size of all Bt crops pooled when compared to non-Bt controls treated with insecticides.
